# Sleep in the age of technology: the use of sleep apps and perceived impact on sleep and sleep habits

**DOI:** 10.3389/fpsyg.2026.1726473

**Published:** 2026-03-20

**Authors:** Håkon Lundekvam Berge, Karl Erik Lundekvam, Siri Waage, Bjørn Bjorvatn, Ståle Pallesen, Ingvild West Saxvig

**Affiliations:** 1 Department of Global Public Health and Primary Care, University of Bergen, Bergen, Norway; 2 Norwegian Competence Center for Sleep Disorders, Haukeland University Hospital, Bergen, Norway; 3 Department of Psychosocial Science, University of Bergen, Bergen, Norway

**Keywords:** digital health, insomnia, orthosomnia, perceived effects, sleep apps, sleep habits, wearable technology, wearables

## Abstract

**Introduction:**

The rapid development in sleep related technology, particularly the emergence of sleep apps, has generated a need to uncover user characteristics and perceived effects.

**Methods:**

A sample of 1,002 adult participants living in Norway completed an online questionnaire on the use and perceived effects of sleep apps (response rate 19.8%). The questions pertained to demographics, including age, sex and educational level in addition to insomnia symptoms assessed with Bergen Insomnia Scale (BIS). The questionnaire also included 10 items focusing on perceived positive and negative effects of sleep app use. Composite scores for negative and positive effects were calculated (range 5–25, with higher scores indicating more negative and positive effects, respectively), and analyzed in relation to different characteristics (age, sex, education, insomnia) using t-tests and one-way ANOVAs. In addition, responses on each item were analyzed in relation to the different characteristics, using chi-square tests and logistic regression analyses.

**Results:**

A total of 46.0% of the participants reported current or previous use of sleep apps. Usage was more prevalent among women and younger individuals (<50 years) but was not associated with educational level or insomnia status. Mean composite score for positive effects was 14.57 ± 4.14, and the mean composite score for negative effects was 10.75 ± 3.85. Neither composite score was associated with sex, whereas both scores varied with age (*p* < 0.001 and *p* = 0.036, respectively), with the youngest respondents scoring highest on both scales. Mean composite scores on negative effects were higher in participants with lower education (*p* = 0.012), and in individuals with insomnia (compared to those without insomnia, *p* < 0.001).

**Conclusion:**

These findings suggest that sleep apps are commonly used in the Norwegian population, and most common among women and younger individuals (<50 years). Younger individuals appeared more responsive to sleep app feedback than older adults, both in terms of positive and negative effects, whereas individuals with lower education seemed more responsive to negative effects specifically. Of note, respondents with insomnia reported more negative effects of sleep app usage, suggesting that it may be wise to caution patients with sleep problems about such use.

## 1 Introduction

In recent years, the use of smartphone health applications (apps) has increased significantly (Krebs and Duncan, 2015; Paradis et al., 2022). These apps often rely on sensors embedded in wearable devices such as smartwatches and fitness bands (Baumert et al., 2022; Chee et al., 2025). This technological progress has coincided with an increased interest and awareness regarding sleep health, and the majority of health apps now include sleep monitoring as part of their daily tracking (Baumert et al., 2022; Shelgikar et al., 2016). Devoted sleep apps are also available with increased attention from the scientific community, as evidenced by the terms Consumer Sleep Technologies, CST (Khosla et al., 2018) and Consumer Health Trackers, CHTs (Chee et al., 2025). Based on input from the different wearable sensors (e.g., accelerometer, pulse- and temperature sensors) often referred to as wearables and pre-set algorithms embedded in the app, sleep apps generally track and report parameters such as sleep latency, sleep duration and sleep efficiency (Bianchi, 2018). Some also report sleep stages and sleep quality, and some may even provide sleep ratings (e.g., sleep score from 0 to 100), as well as suggestions for better sleep and/or diagnostic considerations (Berryhill et al., 2020).

Unfortunately, and despite the rapid increase in sleep app usage, research on sleep app users and their subjective experiences remains limited (Pallesen et al., 2025), not least since wearable sleep tracking is rather new and the technology is rapidly developing. There is also a limited number of scientific studies validating their performance and clinical utility, for example in terms of sleep related breathing disorders (Schneider et al., 2024). Several studies indicate that sleep apps and wearable devices can improve sleep habits by identifying sleep patterns and offering users a better understanding of their sleep (Berryhill et al., 2020; Choi et al., 2018). Still, although sleep apps provide insights into sleep habits, they may also have unintended negative effects. Baron et al. (2017) found that wearables can increase stress and worry about sleep, particularly when the recorded data do not match users’ perceptions or when the apps emphasize sleep deficits or irregularities. This can lead to an excessive focus on the output from the apps, potentially worsening sleep quality (Baron et al., 2017). Sleep app use might lead to orthosomnia (Baron et al., 2017), defined as “excessive preoccupation with sleep” (Guldbrandsen et al., 2025). Research indicate that sleep and sleep habits vary by age and gender (Bjorvatn et al., 2023a; Pallesen et al., 2014; Suzuki et al., 2017). These demographic parameters may influence not only the accuracy of sleep app measurements, but also the reactions to the sleep app output and the subjective experience of usage. Older adults are more disposed to sleep fragmentation and chronic sleep disturbances compared to younger adults (Suzuki et al., 2017), and women report higher rates of insomnia and more fragmented sleep compared to men (Bjorvatn et al., 2023a; Pallesen et al., 2014). Educational level is also associated with sleep. Bjorvatn et al. (2023a,b) found that higher educational level was associated with better sleep health, and it has been reported that individuals with lower education more often experience insomnia symptoms compared to individuals with higher education (Pallesen et al., 2014). Moreover, it is possible that educational level may influence the way reports from sleep apps are interpreted and perceived, but few studies have explored this. Furthermore, it is also plausible that people with sleep problems will react differently to feedback from sleep apps than good sleepers. Insomnia is the most prevalent sleep disorder, characterized by difficulties initiating or maintaining sleep, or early morning awakenings, accompanied by daytime impairments (American Academy of Sleep Medicine, 2023). Individuals with insomnia often exhibit increased sleep-related attentional bias and worry (Harris et al., 2015; Jansson and Linton, 2006) and are more likely to actively engage in various sleep-inducing strategies, including the use of sleep-related apps (Bjorvatn et al., 2023a,b; Ree and Harvey, 2004).

In light of the gap between the growing use, development and availability of sleep apps and the lack of knowledge about how sleep app usage is experienced, it seems important to understand how sleep app users perceive the effects of sleep app use, and to identify individual characteristics (i.e., age, gender, educational level and the presence of sleep problems) that distinguish those who are likely to benefit from those who are not. Such knowledge may guide the customization of sleep apps to better meet individual needs and mitigate possible adverse psychological effects, such as sleep related stress and worry. Thus, the aims of the present study were to: (i) assess the prevalence of sleep app use in the Norwegian adult population, (ii) identify typical user characteristics in terms of sex, age, educational level, and insomnia, and (iii) explore the perceived impact (negative and positive) of sleep apps on sleep and sleep habits, overall and in relation to sex, age, educational level, and insomnia.

## 2 Methods

### 2.1 Procedure

In September 2024, a survey on sleep and sleep habits was conducted among a representative sample of Norwegian adults, aged 18 and older. Data collection was administered by the polling agency Opinion, Oslo, Norway. Targeting 1,000 respondents, the survey was distributed digitally to a total of 5,055 invitees from the “Norstatpanel,” a web panel comprising individuals consenting to take part in research and polls. Participants were informed in advance that the survey included questions on sleep and mental health. In order to obtain a sample reflecting the population, groups with poor response rates were oversampled. A total of 1,002 completed the survey, yielding a response rate of 19.8%. Ethical approval was obtained from the Regional Committee for Medical and Health Research Ethics, South-Eastern Norway (no. 763265).

### 2.2 Sample

The sample included 508 (50.7%) males and 494 women (49.3%), with an average age of 50.3 years (SD = 17.5). A weight factor was calculated, controlling for age, sex and geography, to correct for any imbalances in the sample with respect to the distribution of these variables in the Norwegian population. The weight factor ranged from 0.51 to 2.05 (mean 1.00, median 0.84, standard deviation 0.35). Following weighting adjustment, the sex distribution was 50.1% males and 49.9% females, respectively (see Table 1). The adjusted mean age also declined slightly to 48.8 years (SD = 17.9) following weight adjustment.

**Table 1:** Sample characteristics, and prevalence of sleep app usage in relation to different characteristics (sex, age, education level and insomnia).

Characteristics	Total sample	Sleep app users	Sleep app non-users	*p*-value
	n (% of the sample)	n (% using sleep apps)	n (% not using sleep apps)	Chi-square test
Total sample	1,002 (100.0)	461 (46.0)	540 (53.9)	
Sex				
Male	502 (50.1)	214 (42.6)	288 (57.4)	**0.029**
Female	499 (49.9)	247 (49.5)	252 (50.5)	
Age				
18–35	290 (28.9)	166 (57.2)	124 (42.8)	**<0.001**
36–50	250 (24.9)	143 (57.2)	107 (42.8)	
51–65	233 (23.2)	110 (47.2)	123 (52.8)	
66+	230 (22.9)	42 (18.3)	188 (81.7)	
Higher education*				
No	441 (44.0)	189 (42.9)	252 (57.1)	0.076
Yes	561 (56.0)	272 (48.5)	289 (51.5)	
Insomnia				
No	640 (63.9)	285 (44.5)	355 (55.5)	0.212
Yes	362 (36.1)	176 (48.6)	186 (51.4)	

*No = No academic education at bachelor level or higher, Yes = Academic education at bachelor level or higher.Bold values indicate statistically significant results (*p* < 0.05).

### 2.3 Instruments and questions

#### 2.3.1 Demographics

The polling agency provided information about the respondents sex, age and educational level. For the present study, age was categorized into four groups (18–35, 36–50, 51–65, or 66 + years), whereas educational level was dichotomized (bachelor’s degree or higher, or no higher academic education).

#### 2.3.2 Insomnia symptoms

Insomnia symptoms were assessed using the Bergen Insomnia Scale (BIS) (Pallesen et al., 2008), which was developed based on the diagnostic criteria for insomnia outlined in the Diagnostic and Statistical Manual of Mental Disorders, Fourth Edition (DSM-IV) (American Psychiatric Assosiation, 1994). The scale consists of six items measuring the number of days per week each insomnia symptom is experienced: (i) sleep onset latency (SOL) > 30 min, (ii) wake after sleep onset (WASO) > 30 min, (iii) early morning awakening (EMA) > 30 min before the preferred wake-up time, (iv) non-restorative sleep, (v) daytime impairment, and (vi) dissatisfaction with sleep. Each item is rated on a scale from 0 to 7, based on the number of days per week the symptom is experienced. To conform with the International Classification of Sleep Disorders, third edition, text revision (ICSD-3-TR)/DSM-5, the original BIS time frame of 1 month was modified to assess symptoms over the past 3 months (American Psychiatric Association, 2013; American Academy of Sleep Medicine, 2023). In the present study, the participants were categorized into insomnia and non-insomnia. Insomnia was defined as endorsing (≥3 days per week over the last 3 months) at least one of the first three items (nocturnal insomnia symptoms) as well as at least one of the last two items (daytime symptoms). The BIS has demonstrated acceptable test–retest reliability, as well as good convergent and discriminative validity in relation to other self-report measures and polysomnographic data (Pallesen et al., 2008). The Cronbach’s alpha for the BIS in the current sample was 0.84.

#### 2.3.3 Sleep app use and perceived effect of sleep app use

The participants were asked if they have ever used or are currently using a sleep app (no/yes). Those endorsing that item were then asked which type of sleep app (smartwatch, mobile app, Oura-ring and other devices), they use/used and how they perceived the effects of sleep app use. In the absence of standardized scales on this topic, we administered a questionnaire comprising 10 statements regarding the perceived effects of sleep app use on sleep and sleep habits, inspired by similar research (Pallesen et al., 2025). The first five items focused on experienced positive effects *whereas* the next five focused on experienced negative effects. The responses to these statements were provided on a 5-point Likert scale ranging from 1 (totally disagree), 2 (partly disagree), 3 (neither agree nor disagree), 4 (partly agree), to 5 (totally agree). For the chi-square analyses, responses were categorized into three categories (disagree/totally disagree, neither agree nor disagree, agree/totally agree). For the logistic regression analyses, responses were dichotomized as affirmative (agree, totally agree) or not (disagree, totally disagree, neither agree nor disagree). Two composite scores were also calculated, one for positive and one for negative effects, each ranging from 5 to 25 with higher scores indicating higher positive and negative effects, respectively. Cronbach’s alpha was 0.87 for the positive effects scale and 0.84 for the negative effects scale, indicating high internal consistency.

### 2.4 Statistics

The analyses were conducted using IBM SPSS, version 29.0. Chi-square analyses were used to compare sleep app use and non-usage with respect to sex, age, educational level and insomnia. Focusing on sleep app users only, composite scores for positive and negative effects were compared between the different groups (sex, age, education, insomnia) using t-tests for independent samples and one-way ANOVAs with subsequent LSD *post-hoc* tests. Chi-square analyses were used to compare each of the perceived effects of sleep app use in relation to sex, age, educational level and insomnia. Moreover, logistic regression analyses were conducted to explore odds ratios (OR) and 95% confidence intervals for each of the perceived effects as dependent variables and sex (male sex as reference), age (18–35 years as reference), educational level (not having higher academic education as reference), and insomnia (no insomnia as reference) as independent variables. Both crude and adjusted (all explanatory variables entered simultaneously) models were run.

## 3 Results

Table 1 depicts the characteristics of the study sample in terms of sex, age, educational level and whether they fulfilled the criteria for insomnia, both in the total sample and in relation to sleep app use. A total of 461 (46.0%) respondents confirmed current or previous sleep app use, with most relying on a smartwatch (411), followed by a mobile app (65), other devices (11), and Oura-ring (4). A total of 30 respondents reported having used more than one type of app/device.

Sleep app use was more common amongst females than males (*p* = 0.029). Usage was inversely associated with age (*p* < 0.001), with a usage prevalence of 57.2% in the two youngest age groups (18–35 and 36–50 years) and as few as 18.3% in the oldest age group. There were no significant associations between sleep app use and educational level (higher education yes/no) or insomnia (yes/no) (Table 1).

Mean composite score for positive effects was 14.57 ± 4.14, and the mean composite score for negative effects was 10.75 ± 3.85. There were no significant differences between men and women, neither in terms of positive nor negative effects. Both composite scores varied in relation to age (*p* < 0.001 for positive effects, *p* = 0.036 for negative effects). For positive effects, the youngest participants (18–35 years) scored higher (15.46 ± 3.92) than those aged 51–65 years (13.13 ± 3.84, *p* < 0.001) and 66 + (13.64 ± 3.78, *p* = 0.009), and participants aged 36–50 years scored higher (14.94 ± 4.39) than those aged 51–65 years (13.13 ± 3.84*, p* < 0.001). For negative effects, the youngest participants (18–35 years) reported higher scores (11.24 ± 3.88) than those aged 36–50 years (10.30 ± 3.88, *p* = 0.031) and 66 + (9.66 ± 3.19, *p* = 0.017).

Participants with lower education reported higher composite scores on negative effects (11.28 ± 3.82) compared to those with higher education (10.37 ± 3.83, *p* = 0.012). Individuals with insomnia reported higher composite scores on negative effects (11.63 ± 3.75) than those without insomnia (10.20 ± 3.81, *p* < 0.001).

Table 2 shows how the sleep app users reported each of the single perceived effects. The most frequently reported positive effects (agree or totally agree with the statement) were “*Learned about own sleep”* (48.1%) and “*Perceived usefulness*” (43.5%). “*Improved sleep*” was reported by 15.4%. The most frequently reported negative effects (agree or totally agree with the statement) were “*More worried about my sleep*” (17.8%) and “*Something wrong with my sleep*” (14.0%). “*Worsened sleep*” was reported by 2.3% of the sleep app users.

**Table 2:** How sleep app users (*n* = 461) describe the positive and negative effects of sleep app usage.

Reported effects	n (% of the sample)
Positive effects	
Prioritize sleep: The sleep app made me prioritize sleep more	
Agree/agree very much	63 (29.4)
Neither agree nor disagree	75 (35.0)
Disagree/disagree very much	76 (35.5)
Improved sleep: The sleep app contributed to improve my sleep	
Agree/agree very much	33 (15.4)
Neither agree nor disagree	102 (47.7)
Disagree/disagree very much	79 (36.9)
Improved sleep habits: The sleep app helped me improve my sleep habits	
Agree/agree very much	49 (23.0)
Neither agree nor disagree	88 (35.6)
Disagree/disagree very much	76 (35.7)
Learned about own sleep: I learned a substantial amount about my own sleep through the use of the sleep app	
Agree/agree very much	103 (48.1)
Neither agree nor disagree	66 (30.8)
Disagree/disagree very much	45 (21.0)
Perceived usefulness: I found the sleep app to be very useful	
Agree/agree very much	93 (43.5)
Neither agree nor disagree	80 (37.4)
Disagree/disagree very much	41 (19.2)
Negative effects	
Increased sleep-related stress: The sleep app made me stressed about my sleep	
Agree/agree very much	24 (11.2)
Neither agree nor disagree	52 (24.3)
Disagree/disagree very much	138 (64.5)
Developed concerns about my sleep: Using the sleep app made me think there was something wrong with my sleep	
Agree/agree very much	30 (14.0)
Neither agree nor disagree	55 (25.7)
Disagree/disagree very much	129 (60.3)
More sleep-related worries: The sleep app made me more worried about my sleep	
Agree/agree very much	38 (17.8)
Neither agree nor disagree	59 (27.6)
Disagree/disagree very much	117 (54.7)
Very negative experience: I found the experience of using the sleep app to be very negative	
Agree/agree very much	10 (4.7)
Neither agree nor disagree	46 (21.6)
Disagree/disagree very much	158 (73.8)
Worsened sleep quality: The sleep app use led to worse sleep quality	
Agree/agree very much	5 (2.3)
Neither agree nor disagree	45 (21.0)
Disagree/disagree very much	164 (76.6)

The perceived effects of sleep app use in relation to sex and age are presented in Table 3. There was no significant association between perceived effects of sleep app use and sex, whereas six of the perceived effects were associated with age. These were the positive effects “Prioritize sleep” (*p* < 0.001), “Improved sleep” (*p* = 0.004) and “Improved sleep habits” (*p* < 0.001), and the negative effects “Stressed about my sleep” (*p* = 0.001), “Something wrong with my sleep” (*p* = 0.015) and “More worried about my sleep” (*p* = 0.009). The proportion of respondents agreeing to the statement “Prioritize sleep” ranged from 11.9% in the age group 66 + years to 42.8% in the age group 18–35 years. For the statement “Improved sleep,” the proportion ranged from 5.4% in the age group 51–65 years to 17.6% in the age group 36–50 years, and for the statement “Improved sleep habits” the proportion ranged from 7.1% in the age group 66 + years to 30.1% in the age group 36–50 years. The proportion of respondents agreeing to the statement “Stressed about my sleep” ranged from 2.4% in the age group 66 + years to 22.9% in the age group 18–35 years. For the statement “Something wrong with my sleep” the proportion ranged from 4.8% in the age group 66 + years to 14.7% in the age groups 36–50 years and 51–65 years, and for the statement “More worried about my sleep” the proportion ranged from 4.7% in the age group 66 + years to 23.5% in the age group 18–35 years. See Table 3 for details.

**Table 3:** Perceived effects of sleep app usage in relation to sex and age.

Reported effects	Sex			Age				
	Male	Female	*p*-value	18–35	36–50	51–65	66+	*p*-value
Positive effects								
Prioritize sleep								
Agree/agree very much	63 (29.4)	79 (31.9)	0.745	71 (42.8)	46 (32.2)	20 (18.2)	5 (11.9)	**<0.001**
Neither agree nor disagree	75 (35.0)	79 (31.9)		49 (29.5)	46 (32.2)	41 (37.3)	17 (40.5)	
Disagree/disagree very much	76 (35.5)	90 (36.3)		46 (27.7)	51 (35.7)	49 (44.5)	20 (47.6)	
Improved sleep								
Agree/agree very much	33 (15.4)	28 (11.3)	0.131	26 (15.8)	25 (17.6)	6 (5.4)	3 (7.1)	**0.004**
Neither agree nor disagree	102 (47.7)	107 (43.1)		84 (50.9)	61 (43.0)	44 (39.6)	20 (47.6)	
Disagree/disagree very much	79 (36.9)	113 (45.6)		55 (33.3)	56 (39.4)	61 (55.0)	19 (45.2)	
Improved sleep habits								
Agree/agree very much	49 (23.0)	51 (20.6)	0.211	44 (26.5)	43 (30.1)	11 (10.0)	3 (7.1)	**<0.001**
Neither agree nor disagree	88 (35.6)	88 (35.6)		73 (44.0)	43 (30.1)	38 (34.5)	23 (54.8)	
Disagree/disagree very much	76 (35.7)	108 (43.7)		49 (29.5)	57 (39.9)	61 (55.5)	16 (38.1)	
Learned much about my sleep								
Agree/agree very much	103 (48.1)	117 (47.2)	0.764	83 (50.0)	72 (50.3)	45 (40.9)	20 (46.5)	0.427
Neither agree nor disagree	66 (30.8)	72 (29.0)		53 (31.9)	38 (26.6)	33 (30.0)	14 (32.6)	
Disagree/disagree very much	45 (21.0)	59 (23.8)		30 (18.1)	33 (23.1)	32 (29.1)	9 (20.9)	
Very useful								
Agree/agree very much	93 (43.5)	97 (39.1)	0.304	68 (41.2)	71 (49.7)	33 (30.0)	17 (40.5)	0.052
Neither agree nor disagree	80 (37.4)	91 (36.7)		65 (39.4)	42 (29.4)	46 (41.8)	18 (42.9)	
Disagree/disagree very much	41 (19.2)	60 (24.2)		32 (19.4)	30 (21.0)	31 (28.2)	7 (16.7)	
Negative effects								
Stressed about my sleep								
Agree/agree very much	24 (11.2)	38 (15.4)	0.304	38 (22.9)	11 (7.7)	12 (10.8)	1 (2.4)	**0.001**
Neither agree nor disagree	52 (24.3)	65 (26.3)		37 (22.3)	37 (25.9)	31 (27.9)	13 (31.0)	
Disagree/disagree very much	138 (64.5)	144 (58.3)		91 (54.8)	95 (66.4)	68 (61.3)	28 (66.7)	
Something wrong with my sleep								
Agree/agree very much	30 (14.0)	33 (13.3)	0.677	23 (13.9)	21 (14.7)	16 (14.7)	2 (4.8)	**0.015**
Neither agree nor disagree	55 (25.7)	56 (22.6)		44 (26.5)	26 (18.2)	35 (32.1)	5 (11.9)	
Disagree/disagree very much	129 (60.3)	159 (64.1)		99 (59.4)	96 (67.1)	58 (53.2)	35 (83.3)	
More worried about my sleep								
Agree/agree very much	38 (17.8)	38 (15.4)	0.734	39 (23.5)	18 (12.6)	17 (15.5)	2 (4.7)	**0.009**
Neither agree nor disagree	59 (27.6)	74 (30.0)		50 (30.1)	40 (28.0)	34 (30.9)	9 (20.9)	
Disagree/disagree very much	117 (54.7)	135 (54.7)		77 (46.4)	85 (59.4)	59 (53.6)	32 (74.4)	
My experience was very negative								
Agree/agree very much	10 (4.7)	10 (4.0)	0.171	7 (4.2)	5 (3.5)	7 (6.4)	1 (2.4)	0.509
Neither agree nor disagree	46 (21.6)	72 (29.1)		42 (25.3)	31 (21.5)	32 (29.1)	14 (33.3)	
Disagree/disagree very much	158 (73.8)	165 (66.8)		117(70.5)	108 (75.0)	71 (64.5)	27 (64.3)	
Led to worse sleep quality								
Agree/agree very much	5 (2.3)	7 (2.8)	0.943	5 (3.0)	4 (2.8)	2 (1.8)	0 (0.0)	0.592
Neither agree nor disagree	45 (21.0)	51 (20.6)		31 (18.8)	26 (18.2)	29 (26.4)	10 (23.8)	
Disagree/disagree very much	164 (76.6)	189 (76.5)		129 (78.2)	113 (79.0)	79 (71.8)	32 (76.2)	

Bold values indicate statistically significant results (*p* < 0.05).

Table 4 depicts the perceived positive and negative effects of sleep app use in relation to educational level and insomnia. No significant associations were found between perceived effects of sleep app use and education. However, there were significant associations between some of the perceived negative effects of sleep app use and insomnia. Participants with insomnia were more inclined to agree/totally agree with the statements “*Something wrong with my sleep*” (*p* = 0.013) and “*More worried about my sleep*” (*p* < 0.001), but less inclined to agree/totally agree with the statement “*Led to worse sleep quality*” (*p* = 0.014).

**Table 4:** Perceived effects of sleep app usage in relation to educational level and insomnia.

Reported effects	Higher education			Insomnia		
	No	Yes	*p*-value	No	Yes	*p*-value
Positive effects						
Prioritize sleep						
Agree/agree very much	48 (25.3)	94 (34.6)	0.102	87 (30.5)	54 (30.7)	0.614
Neither agree nor disagree	69 (36.3)	85 (31.3)		91 (31.9)	63 (35.8)	
Disagree/disagree very much	73 (38.4)	93 (24.2)		107 (37.5)	59 (33.5)	
Improved sleep						
Agree/agree very much	27 (14.3)	33 (12.1)	0.127	36 (12.6)	25 (14.2)	0.166
Neither agree nor disagree	75 (39.7)	134 (49.3)		139 (48.8)	70 (39.8)	
Disagree/disagree very much	87 (46.0)	105 (38.6)		110 (38.6)	81 (46.0)	
Improved sleep habits						
Agree/agree very much	40 (21.1)	61 (22.4)	0.896	67 (23.5)	34 (19.2)	0.554
Neither agree nor disagree	75 (39.5)	102 (47.5)		107 (37.5)	70 (39.5)	
Disagree/disagree very much	75 (39.5)	109 (40.1)		111 (38.9)	73 (41.2)	
Learned much about my sleep						
Agree/agree very much	89 (46.8)	132 (48.5)	0.859	143 (50.4)	77 (43.8)	0.310
Neither agree nor disagree	59 (31.1)	78 (28.7)		78 (27.5)	59 (33.5)	
Disagree/disagree very much	42 (22.1)	62 (22.8)		63 (22.2)	40 (22.7)	
Very useful						
Agree/agree very much	69 (36.5)	120 (44.3)	0.159	122 (42.8)	67 (37.9)	0.435
Neither agree nor disagree	79 (41.8)	91 (33.6)		105 (36.8)	66 (37.3)	
Disagree/disagree very much	41 (21.7)	60 (22.1)		58 (20.4)	44 (24.9)	
Negative effects						
Stressed about my sleep						
Agree/agree very much	26 (13.7)	36 (13.2)	0.100	30 (10.5)	32 (18.2)	0.060
Neither agree nor disagree	58 (30.5)	60 (22.1)		73 (25.6)	44 (25.0)	
Disagree/disagree very much	106 (55.8)	176 (64.7)		182 (63.9)	100 (56.8)	
Something wrong with my sleep						
Agree/agree very much	22 (11.6)	40 (14.8)	0.259	37 (13.0)	26 (14.8)	**0.013**
Neither agree nor disagree	52 (27.5)	58 (21.4)		56 (19.6)	54 (30.7)	
Disagree/disagree very much	115 (60.8)	173 (63.8)		192 (67.4)	96 (54.5)	
More worried about my sleep						
Agree/agree very much	31 (16.4)	44 (16.2)	0.257	41 (14.4)	34 (19.3)	**<0.001**
Neither agree nor disagree	62 (32.8)	71 (26.1)		64 (22.5)	69 (39.2)	
Disagree/disagree very much	96 (50.8)	157 (57.7)		179 (63.0)	73 (41.5)	
Very negative experience						
Agree/agree very much	13 (6.8)	7 (2.6)	0.065	9 (3.2)	11 (6.2)	0.186
Neither agree nor disagree	51 (26.8)	68 (25.0)		70 (24.6)	49 (27.7)	
Disagree/disagree very much	126 (66.3)	197 (72.4)		206 (72.3)	117 (66.1)	
Led to worse sleep quality						
Agree/agree very much	4 (2.1)	8 (2.9)	0.276	8 (2.8)	3 (2.3)	**0.014**
Neither agree nor disagree	46 (24.3)	50 (18.4)		47 (16.5)	49 (27.8)	
Disagree/disagree very much	139 (73.5)	214 (78.7)		230 (80.7)	123 (69.9)	

Table 5 shows results from the crude and adjusted logistic regression analyses, exploring the perceived positive and negative effects of sleep app use in relation to sex, age, education and insomnia. Sex was not associated with perceived effects of sleep app use, neither in the crude nor in the adjusted analyses. In terms of positive effects, older age groups were associated with reduced likelihood of agreeing/totally agreeing with the statement “*Prioritize sleep*,” (OR 0.57, 0.29, and 0.16, respectively in the adjusted analysis). Age between 36 and 50 years was associated with reduced likelihood of reporting “*Better sleep*” (OR 0.29 in the adjusted analyses), whereas the two oldest age group were associated with reduced likelihood of reporting “*Improved sleep habits*” (OR 0.32 and 0.20 respectively, in the adjusted analyses). In terms of negative effects, older age was associated with reduced likelihood of being “*Stressed about sleep*” (OR 0.29, 0.40, and 0.08, respectively). Age groups 36–50 and 66 + were also associated with reduced likelihood for reporting “*More worried*” (OR 0.45 and 0.13 respectively). Higher education was associated with higher likelihood of agreeing/totally agreeing with the statement “*Prioritize sleep*” (OR 1.72 in the adjusted analyses), but no other associations were found between educational level and perceived effects of sleep app use in the adjusted analyses. Insomnia was not associated with perceived effects of sleep app use in any of the adjusted analyses.

**Table 5:** Crude and adjusted logistic regression analyses with the perceived effects of sleep app use (totally disagree/disagree/neutral = 0; agree/totally agree = 1) as the dependent variable and sex, age, higher education and insomnia as predictors (*n* = 461).

Characteristics	Positive effects									
	Prioritize sleep		Better sleep		Improved sleep habits		Learned about my sleep		Very useful	
	Crude	Adjusted	Crude	Adjusted	Crude	Adjusted	Crude	Adjusted	Crude	Adjusted
Sex										
Male	1.00 (ref)	1 (ref)	1 (ref)	1 (ref)	1 (ref)	1 (ref)	1 (ref)	1 (ref)	1 (ref)	1 (ref)
Female	1.12 (0.76–1.67)	1.07 (0.70–1.63)	0.69 (0.40–1.19)	0.72 (0.41–1.27)	0.87 (0.56–1.36)	0.94 (0.59–1.49)	0.96 (0.66–1.38)	1.00 (0.69–1.46)	0.84 (0.58–1.22)	0.87 (0.59–1.28)
Age										
18–35	1 (ref)	1 (ref)	1 (ref)	1 (ref)	1 (ref)	1 (ref)	1 (ref)	1 (ref)	1 (ref)	1 (ref)
36–50	0.64 (0.4–1.01)	**0.57** **(0.35–0.92)**	1.15 (0.63–2.10)	1.22 (0.65–2.28)	1.91 (0.73–1.96)	1.13 (0.68–1.89)	1.00 (0.64–1.57)	0.95 (0.60–1.51)	1.40 (0.89–2.19)	1.29 (0.81–2.04)
51–65	**0.30** **(0.17–0.53)**	**0.29** **(0.16–0.51)**	**0.28** **(0.11–0.73)**	**0.29** **(0.11–0.74)**	**0.32** **(0.16–0.64)**	**0.32** **(0.16–0.64)**	0.69 (0.43–1.12)	0.69 (0.42–1.13)	0.61 (0.37–1.02)	0.61 (0.37–1.02)
66+	**0.18** **(0.07–0.49)**	**0.16** **(0.06–0.44)**	0.43 (0.13–1.45)	0.46 (0.13–1.60)	**0.23** **(0.07–0.75)**	**0.20** **(0.06–0.68)**	0.85 (0.43–1.68)	0.78 (0.39–1.55)	0.99 (0.50–1.97)	0.91 (0.45–1.83)
Higher education										
No	1 (ref)	1 (ref)	1 (ref)	1 (ref)	1 (ref)	1 (ref)	1 (ref)	1 (ref)	1 (ref)	1 (ref)
Yes	**1.59** **(1.05–2.40)**	**1.72** **(1.11–2.66)**	0.83 (0.48–1.42)	0.79 (0.45–1.40)	1.09 (0.70–1.72)	1.03 (0.64–1.65)	1.07 (0.74–1.55)	1.04 (0.71–1.53)	1.39 (0.95–2.03)	1.31 (0.89–1.95)
Insomnia										
No	1 (ref)	1 (ref)	1 (ref)	1 (ref)	1 (ref)	1 (ref)	1 (ref)	1 (ref)	1 (ref)	1 (ref)
Yes	1.01 (0.68–1.52)	0.93 (0.61–1.44)	1.16 (0.67–2.01)	1.25 (0.70–2.23)	0.78 (0.49–1.23)	0.76 (0.47–1.24)	0.76 (0.52–1.11)	0.77 (0.52–1.13)	0.81 (0.55–1.20)	0.89 (0.60–1.34)

Characteristics	Negative effects									
	Stressed about sleep		Something wrong with my sleep		More worried		Negative experience		Worse sleep quality	
	Crude	Adjusted	Crude	Adjusted	Crude	Adjusted	Crude	Adjusted	Crude	Adjusted
Sex										
Male	1 (ref)	1 (ref)	1 (ref)	1 (ref)	1 (ref)	1 (ref)	1 (ref)	1 (ref)	1 (ref)	1 (ref)
Female	1.48 (0.85–2.55)	1.28 (0.72–2.27)	0.93 (0.55–1.58)	0.87 (0.50–1.50)	0.84 (0.51–1.38)	0.74 (0.45–1.24)	0.84 (0.34–2.07)	0.79 (0.31–1.99)	1.40 (0.43–4.56)	1.44 (0.43–4.82)
Age										
18–35	1 (ref)	1 (ref)	1 (ref)	1 (ref)	1 (ref)	1 (ref)	1 (ref)	1 (ref)	1 (ref)	1 (ref)
36–50	**0.28** **(0.14–0.57)**	**0.29** **(0.14–0.61)**	1.06 (0.56–2.00)	1.00 (0.52–1.92)	**0.46** **(0.25–0.84)**	**0.45** **(0.24–0.84)**	0.70 (0.21–2.32)	0.95 (0.27–3.30)	0.90 (0.52–3.27)	0.85 (0.23–3.17)
51–65	**0.41** **(0.20–0.82)**	**0.40** **(0.20–0.81)**	1.07 (0.54–2.12)	1.07 (0.54–2.12)	0.59 (0.32–1.11)	0.60 (0.32–1.12)	1.51 (0.52–4.33)	1.58 (0.54–4.59)	0.62 (0.13–2.99)	0.62 (0.13–2.96)
66+	**0.07** **(0.01–0.60)**	**0.08** **(0.01–0.70)**	0.25 (0.05–1.24)	0.24 (0.05–1.13)	**0.13** **(0.03–0.65)**	**0.13** **(0.03–0.67)**	0.42 (0.04–4.27)	0.58 (0.05–6.24)	**0.00** **(0.00–0.00)**	**0.00** **(0.00–0.00)**
Higher education										
No	1 (ref)	1 (ref)	1 (ref)	1 (ref)	1 (ref)	1 (ref)	1 (ref)	1 (ref)	1 (ref)	1 (ref)
Yes	0.97 (0.57–1.68)	1.21 (0.68–2.15)	1.31 (0.75–2.28)	1.38 (0.78–2.44)	0.97 (0.59–1.61)	1.17 (0.69–1.98)	**0.37** **(0.14–0.93)**	0.41 (0.16–1.07)	1.20 (0.36–3.94)	1.14 (0.34–3.86)
Insomnia										
No	1 (ref)	1 (ref)	1 (ref)	1 (ref)	1 (ref)	1 (ref)	1 (ref)	1 (ref)	1 (ref)	1 (ref)
Yes	**1.90** **(1.11–3.26)**	1.56 (0.89–2.74)	1.17 (0.68–2.01)	1.12 (0.64–1.96)	1.45 (0.88–2.38)	1.28 (0.77–2.16)	1.95 (0.80–4.79)	1.71 (0.66–4.42)	0.68 (0.19–2.38)	0.59 (0.16–2.12)

In the adjusted model, all predictors were entered in the same analysis. Results are reported as odds ratio and 95% confidence interval.Bold values indicate statistically significant results (*p* < 0.05).

## 4 Discussion

Nearly half of the participants used or had previously used sleep apps, with usage being more common among women and younger adults. The most frequently reported positive effects of sleep app use were “*Learning about own sleep*” and “*Perceived usefulness*,” while few reported “*Improved sleep quality*.” Negative effects were overall reported less frequently, with “*More sleep-related worries*” and “*Developed concerns about my sleep*” being the most commonly reported, whereas “*Worsened sleep quality*” was rarely reported. Younger age was a significant predictor for several of the reported effects of sleep app use, both positive and negative, whereas higher education was associated with “*Prioritize sleep*.”

### 4.1 Sleep app use

The use of wearable health care devices has increased significantly in recent years, driven by advances in sensor technology and growing consumer interest (Chandrasekaran et al., 2020; Chee et al., 2025). Sleep apps have generally exhibited poor accuracy in relation to both subjective sleep experience and polysomnography, the latter known as the gold standard within diagnostic sleep measurement (Al Mahmud et al., 2022; Bhat et al., 2015). This extends not only to sleep–wake measurements and sleep stages, but also identification of sleep related events, such as sleep related breathing abnormalities (Trecca et al., 2021; Trecca et al., 2024). Sleep apps should therefore not replace validated diagnostic sleep assessment, such as polysomnography (Choi et al., 2018), although a role for sleep apps in preliminary screening for sleep disorders has been suggested (Trecca et al., 2024). Existing research indicates that the use of health apps is more prevalent among younger individuals than among older adults (Carroll et al., 2017), and in a primary care study from 2022, 47.7% of smartphone users had used at least one health app, with significantly higher use among individuals below 30 years (Paradis et al., 2022). The present study seems to support such notions, with ~60% of respondents in the two youngest age groups (18–35 and 36–50) reporting sleep app use, compared to ~50% and ~20% in the two oldest age groups, respectively. Contrasting young adults’ high engagement with health apps (Peng et al., 2016), lower usage among older adults may be influenced by barriers related to usability and technology acceptance in general (Zaman et al., 2022), in addition to the fact that most sleep apps are designed for younger age groups (Shelgikar et al., 2016). In the present study we also found that women reported higher usage rates of sleep apps than men. Existing research shows that women are more likely to prefer apps related to nutrition, self-care, and reproductive health, whereas men generally show a preference for fitness-related apps (Bol et al., 2018; Wang and Qi, 2021). This may offer one explanation of women’s increased use of sleep functions in the health apps.

Interestingly, educational level was not associated with sleep app use. Higher education is shown to be associated with increased use of health technologies, including sleep apps, as individuals with more education and those with high health literacy are more likely to adopt such tools (Jacob et al., 2022; Nagappan et al., 2024; Tuitert et al., 2024). A recent systematic review on digital health literacy (DHL), defined as “the ability to find, understand and use health information from digital sources” showed that education is positively associated with DHL (Estrela et al., 2023). However, the relationship is not always straightforward, and some studies indicate that factors such as income, age, and overall health awareness also play a significant role (Bol et al., 2018; Robbins et al., 2020).

An unexpected finding in the present study was the absence of associations between sleep app use and insomnia. Previous research has suggested that individuals experiencing sleep disturbances are more likely to engage in self-monitoring behaviors to regulate their sleep patterns (Huberty et al., 2019). For example, in a study by Aji et al. (2019), 93% of individuals with sleep disturbances expressed willingness to use an evidence-based sleep application to improve their sleep. Similarly, a large survey of young adults (18–30 years) found that more than one-third of participants had adopted sleep applications, with usage being significantly higher among individuals with more pronounced insomnia symptoms (Pallesen et al., 2025). Given the high prevalence of digital sleep-tracking behaviors among those with sleep difficulties, we anticipated a positive association between sleep app use and insomnia. One explanation for the lack of an association between sleep app use and insomnia symptoms in our study may be that sleep app use was motivated by general health awareness and curiosity, rather than actual clinical sleep disturbances. Another possible explanation for the insignificant findings can be that individuals with insomnia might prefer evidence-based interventions over self-tracking apps. Future research should investigate the psychological drivers of sleep app use, particularly among individuals with sleep disorders.

### 4.2 Perceived effects of sleep app use

Overall, participants reported both positive and negative effects of sleep app use, but composite scores revealed that positive effects were more often reported than negative. These findings align with previous research suggesting that wearable sleep tracking devices can enhance sleep awareness, but may also contribute to increased sleep-related stress/worry (Karasneh et al., 2022; Van Den Bulck, 2015). The findings are also consistent with prior research indicating that health apps can, in some cases, provide users with a misleading sense of reassurance about their sleep and these apps may also heighten anxiety by emphasizing minor variations in sleep data that are not clinically significant, causing unnecessary concern and stress among users (Baron et al., 2017).

Age was the most influential factor in shaping users’ experiences with sleep apps. Our findings may indicate that younger users are more strongly affected, both positively and negatively, by sleep app use compared to older adults. Specifically, younger age groups (18–35 and 36–50 years) were more likely to report beneficial effects, such as “*Improved sleep*” and “*Prioritize sleep*,” whereas older participants (66+) were less likely to report such advantages. Younger adults have a greater tendency to use electronic media in bed (Bjorvatn et al., 2017), which suggests that younger age groups may have more potential to improve their sleep habits, and thus report greater perceived effects from sleep apps. Moreover, the two youngest age groups also reported higher levels of stress and concern (“*Increased sleep-related stress*” and “*Developed concerns about my sleep”*), suggesting that they may be more susceptible to the negative effects of digital health information. Possible explanations might be related to younger adults’ superior engagement in health apps (Peng et al., 2016) and that they are more likely to explore the different features of health apps (Kim and Choudhury, 2020). In comparison, older adults more frequently struggle to adapt to rapid changes in technology (Pirhonen et al., 2020). Furthermore, Kebede et al. (2022) found that older adults’ lack of capability and motivation was among the main contributing factors to their inferior digital engagement. Despite women reporting more sleep app use than men, there were no significant sex differences in perceived effects of usage. The composite score analyses as well as the regression models showed that men and women reported the impact of sleep apps in a relatively similar manner. This indicates that sex is not a strong predictor of how users experience the effects of sleep apps. Regarding the impact of education, participants with lower education reported higher composite scores on negative effects compared to those with higher education, which may indicate that this group is more vulnerable to worry and stress elicited by to sleep app use. Research shows that individuals with higher education are more likely to seek information before purchasing products (Besler et al., 2012), which may make them more critical and informed users in general. Furthermore, Pallesen et al. (2025) found that individuals with higher education reported fewer positive effects when using sleep apps than those with lower education.

The composite score analyses indicated a general tendency for individuals with insomnia to report more negative effects of sleep app use than individuals without insomnia. In particular, individuals with insomnia seemed to be more prone to negative thoughts and worries about sleep, as suggested by results from the chi-square analyses showing that individuals with insomnia more often agreed/totally agreed with the statements “*Something wrong with my sleep*” and “*More worried about my sleep*,” whereas they were less inclined to agree/totally agree with the statement “*Led to worse sleep quality*.” Several mechanisms may explain this heightened vulnerability. Individuals with insomnia often exhibit excessive cognitive activity and attentional biases (e.g., heightened focus on sleep-related threats and negative expectations about sleep interventions) (Harvey, 2002), which would theoretically predispose them to report more negative effects from sleep app use (Harris et al., 2015). Dysfunctional beliefs about sleep, combined with persistent arousal, can further contribute to the maintenance of insomnia symptoms (Jansson and Linton, 2006). Difficulties disengaging from sleep-related concerns may further influence how individuals with insomnia respond to and engage with app-based tools (Fabbri et al., 2022). As a result, sleep app use may serve as a potential contributor to increased sleep-related stress and worry (Baron et al., 2017). Of note, the findings from the chi-square analyses were not supported by the adjusted logistic regression analyses, in which insomnia did not increase the odds for reporting any particular perceived effect. Thus, the effect of sleep app usage in relation to insomnia needs to be further explored in future studies. Taken together, the present findings have important implications. Sleep apps may need to be tailored more specifically to individuals with sleep disorders, potentially incorporating evidence-based interventions to better address their needs.

#### 4.2.1 Strengths and limitations

The present study is based on a large sample intended to reflect the most important characteristics of the Norwegian population. The respondents were drawn from a web panel consisting of potential respondents with a wide range of age and educational level. Web panels, however, cannot be regarded as representative because the participants are not randomly selected, but rather self-selected. Therefore, individuals with more leisure, interest in digital surveys or the survey rewards might be more inclined to participate. Weighting of the data were applied to correct for discrepancies between the population and the distribution of sex, age, and educational level. A validated insomnia scale, BIS (Pallesen et al., 2008), was used. This scale is based on the criteria outlined in DSM-5/ICSD-3 (American Academy of Sleep Medicine, 2023; American Psychiatric Association, 2013). Still, this categorization was based solely on data collected through a questionnaire, and not clinical interviews. The problem with relying only on BIS is that a questionnaire alone cannot exclude the possibility that the insomnia-like symptoms may reflect other underlying issues, such as circadian rhythm sleep–wake disorders or obstructive sleep apnea (Bjorvatn et al., 2021). Regression analyses were conducted to adjust for confounding variables, thus providing a better estimate of the association of each variable. The nature of explorative studies, as the present one, also represents some limitations to consider (e.g., not testing *a priori* presented hypotheses). A high amount of significance tests increases the risk of type 1 error, which might yield false positive results (Barnett et al., 2022). Another limitation of the present study is the low response rate (19.8%), which may indicate selection bias. Because the invitation stated that the survey would include questions on sleep and mental health, it is possible that individuals with experiences or concerns in these areas were more inclined to respond. This could potentially affect the generalizability of the reported prevalences. Still, such low response rates are not uncommon and align with response rates in similar population-based studies (Bjorvatn et al., 2023a; Morin et al., 2006). The items exploring the subjective user effects were not validated, but constructed in accordance with the existing, but limited research on positive and negative experiences of sleep apps (Pallesen et al., 2025). All the data were based on self-reports in a descriptive cross-sectional study design. Consequently, the findings may have been influenced by factors like recall bias (Talari and Goyal, 2020) and common method bias, the latter implying skewing of results when data are solely derived from a single data source (Podsakoff et al., 2003). Furthermore, sleep app use was assessed in terms of life-time use, hence nuances regarding duration and intensity of use were not taken into consideration. Therefore, it might have been more purposeful to rephrase the question to only include participants who had used sleep functions over an extended amount of time. Awareness of these limitations is vital for interpreting the results (Talari and Goyal, 2020). Furthermore, cross-sectional study designs are not suited for drawing conclusions about directionality and causality, hence future experimental studies are warranted. Also, studies investigating which sleep app functions are being used and perceived impact of these should also be conducted.

## 5 Conclusion

Results from the present study showed that sleep apps were commonly used, and that the perceived effects of usage depended on individual characteristics. Future research should focus more on the objective effects of sleep apps, preferably through polysomnography to obtain validated sleep (quality and duration) measurements. It would also be beneficial to employ longitudinal or experimental designs to draw conclusions on directionality and causality on this topic. Importantly, the effect of sleep app usage in relation to insomnia needs to be further explored in future studies. Moreover, future research should explore the long-term psychological and behavioral effects of sleep app use, particularly in relation to stress and overall sleep health and also the reasons provided by uses for initiating and stop using.

## Data Availability

The original contributions presented in the study are included in the article/supplementary material, further inquiries can be directed to the corresponding authors.
